# Co-encapsulated Ce6 + CpG and biopeptide-modified liposomes for enhanced transdermal photo-immunotherapy of superficial tumors

**DOI:** 10.1016/j.mtbio.2025.101669

**Published:** 2025-03-13

**Authors:** Shaozhen Wang, Chen Yang, Yuanyuan Zhang, Yi Hu, Lan Xiao, Weiping Ding, Bensheng Qiu, Fenfen Li

**Affiliations:** a Medical Imaging Center, Department of Electronic Engineering and Information Science, University of Science and Technology of China, Hefei, Anhui, 230026, China; b School of Biomedical Engineering, Division of Life Sciences and Medicine, University of Science and Technology of China, Hefei, Anhui, 230026, China; c Suzhou Institute for Advanced Research, University of Science and Technology of China, Suzhou, 215123, China; d Department of Burns, The First Affiliated Hospital of Anhui Medical University, Hefei, Anhui, 230027, China; e Department of Gynecology Oncology, The First Affiliated Hospital of USTC, Division of Life Sciences and Medicine, University of Science and Technology of China, Hefei, Anhui, 230031, China; f Department of Oncology, The First Affiliated Hospital of USTC, Division of Life Sciences and Medicine, University of Science and Technology of China, Hefei, Anhui, 230001, China

**Keywords:** Transdermal-enhancing peptide, Transdermal delivery, Cationic liposomes, Superficial tumors, Photo-immunotherapy

## Abstract

Cancer immunotherapy encounters challenges of a low treatment response rate due to the tumor immunosuppressive microenvironment and immune-related adverse events caused by off-target immunotherapy agents delivered through systemic administration in clinical practice. Photodynamic therapy (PDT) offers a viable approach to improve the immunotherapy efficacy through inducing immunogenic tumor cell death and is particularly advantageous in superficial tumor treatment. Therefore, leveraging integrated nanomaterials for photo-immunotherapy appears to be an ideal strategy to improve therapeutic outcome. In this study, we develop a transdermal-enhancing peptide (TD)-modified cationic liposome that simultaneously encapsulated with photosensitizer chlorine 6 (Ce6) and immunoadjuvant CpG, denoted as Ce6/CpG@Lip-TD, to mediate photo-immunotherapy of superficial tumors via the skin. The functionalization of TD peptide and positively charged surface endow the liposomes enhanced skin penetration capability. The combination of Ce6 and CpG within the liposomes synergistically potentiates the photo-immunotherapy effect when exposed to laser irradiation. In both melanoma and breast cancer murine models, Ce6/CpG@Lip-TD demonstrated substantial tumor-suppressing properties, along with an augmented systemic immune response against distal tumors. As a topical therapeutic agent, Ce6/CpG@Lip-TD circumvents the regulatory challenges associated with the systemic delivery of nanomaterials and significantly reduces systemic side effects, holding great promise for rapid translation into clinical applications.

## Highlights

• Ce6 and CpG co-loaded liposomes are developed for tumor photo-immunotherapy.
• TD peptide significantly enhances the skin penetration ability of liposomes.
• The liposomes effectively induce ICD and subsequent systemic antitumor immunity.
• The liposomes inhibit tumor growth in both melanoma and breast tumor murine models.

## Graphical abstract

**Figure:**
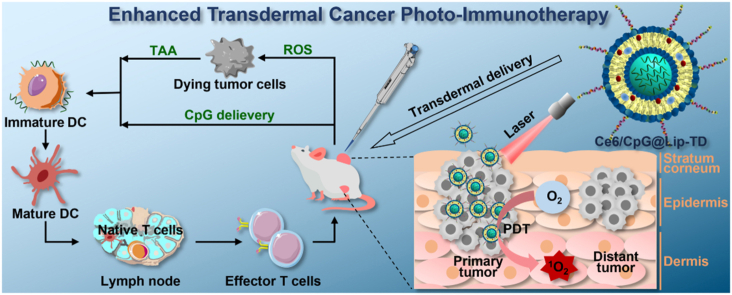

## 1 Introduction

Cancer has become a prevalent public health concern worldwide and is expected to surpass cardiovascular disease and become the leading cause of premature death in most countries [1,2]. Superficial malignant tumors, including melanoma, T lymphocyte carcinoma, basal cell carcinoma and squamous cell carcinoma of the skin, pose a serious threat to human life and health [[3], [4], [5]]. While surgical excision traditionally serves as the principal therapeutic intervention for these superficial tumors, its implementation is often hindered by significant invasiveness, risk of wound infections, and propensity for recurrence. Furthermore, it typically falls short in eliciting a systemic anti-tumor response to inhibit metastasis. Besides surgery, current therapeutic approaches like chemotherapy, radiation therapy, photodynamic/photothermal therapy, immunotherapy, targeted therapy and combination therapy have been utilized in combatting cancers [6]. Immunotherapy, in particular, has emerged as a powerful tool in the field of oncology, leveraging the intricate host immune system to identify and eliminate tumors [[7], [8], [9]]. Despite it's great success, particularly in melanoma treatment, a substantial proportion of melanoma patients remain unresponsive to immunotherapy, primarily attributed to the existence of a tumor immunosuppressive microenvironment [10]. Additionally, immune-related adverse events (irAEs) such as endocrine diseases, enteritis, hepatitis, etc., caused by off-target effects due to systemic administration of immune stimulating molecules, present a formidable clinical challenge [11,12]. Consequently, there is an urgent need to explore innovative combinatorial immunotherapeutic strategies and develop targeted drug delivery systems to enhance the therapeutic efficacy while minimize adverse effects in the management of cancer.

Photodynamic therapy (PDT) involves irradiating a photosensitizer with a light source, triggering the generation of singlet oxygen (^1^O_2_) from the surrounding oxygen molecules, which efficaciously exterminates cancer cells [13]. PDT has garnered significant interest as a treatment for a variety of diseases, particularly for superficial tumors. This is due to its precise spatiotemporal control, noninvasive nature, and exceptional selectivity for target tissues [14]. Chlorin e6 (Ce6), an FDA-approved photosensitizer, is known for its high quantum yield of singlet oxygen and its favorable biocompatibility [15]. Nonetheless, like most photosensitizers, Ce6 is aromatic and hydrophobic in nature with poor or limited solubility in aqueous environments [16,17]. The encapsulation of photosensitizers within water-dispersible nanocarriers to form a nanoparticle formulation is a promising strategy to improve their solubility, thereby facilitating their application in anti-tumor therapeutics [18]. Apart from directly eradicating cancer cells, PDT can also trigger immunogenic cell death (ICD), prompting the exposure of damage-associated molecular patterns (DAMPs) to initiate an anti-tumor immune response [19]. To enhance the anticancer efficiency and amplify the in situ photo-immune response, various immunoadjuvants or immune checkpoint inhibitors have been simultaneously incorporated into a single nanomaterial, including Toll-like receptor (TLR) agonists like CpG [20], R848 [21] and R837 [22]; inorganic immunoadjuvants like alum [23] and magnesium [24,25]; as well as immune checkpoint inhibitors like CTLA-4, PD-1 or PD-L1 blocking antibodies [26]. Among them, the CpG oligodeoxynucleotides can enhance anti-tumor effect by activating TLR9 extensively expressed in both murine and human dendritic cells (DCs) and initiate a signaling cascade of innate and adaptive immune responses, rendering it an efficacious immunoadjuvant [27]. For example, Chen et al. reported a CpG-loaded multifunctional nanoplatform (FA-CuS/DTX@PEI-PpIX-CpG nanocomposites) for synergistic phototherapy and docetaxel (DTX)-enhanced immunotherapy to treat triple negative breast cancers [28]. Ni et al. designed a cationic nanoscale metal-organic framework (W-TBP) capable of adsorbing anionic CpG to obtain W-TBP/CpG, facilitating tumor antigen presentation by enabling immunogenic PDT and promoting the maturation of DCs [29]. Therefore, employing nanocarriers for the delivery of Ce6 and CpG represents an effective strategy for enhancing tumor photoimmunotherapy. Indeed, the combination of Ce6 and CpG for tumor photoimmunotherapy has been explored previously, with various carriers being utilized for co-delivery, such as metal-organic frameworks (MOFs) [30], TiO_2_ nanoparticles [31],polydopamine stabilized graphene quantum dots (PC@GCpD(Gd)) [32], mesoporous silica nanoparticles (MSN) [33,34] and human heavy chain ferritin (HFn) [35]. However, systemic administration of these nanomaterials may lead to less than ∼1 % of the nanoparticles being enriched at the tumor site, with the major proportion accumulating in the liver, causing systematic side effect [36].

For the management of cutaneous malignancies, the utilization of transdermal drug delivery has emerged as a prominent method of localized drug administration, attracting extensive interest due to its non-invasive nature, patient compliance, heightened bioavailability, and minimal systemic toxicity [37,38]. However, the stratum corneum, as the first protective barrier of the human body, poses a significant hindrance to the efficient transdermal transport of most therapeutic agents. Employing nanocarriers has emerged as a viable approach to surmount the stratum corneum barrier without inducing tissue damage, thereby facilitating effective drug penetration. Various nanocarriers, such as liposomes [39], inorganic nanoparticles [40], nano/micro-emulsions [41], nanogels [42], dendrimers [43], and others, have been extensively investigated for their potential in this realm. Among these nanocarriers investigated, liposomal nanocarriers exhibit obvious advantages for transdermal drug delivery, characterized by their structural resemblance to cellular membranes, controlled drug release, augmented absorption, and diminished adverse effects [44]. Additionally, many liposome-based nanocarriers have received approval from the FDA, such as Doxil®, Onivyde®, Vyxeos®, and Onpattro® [45], underscoring their clinical relevance and safety profile. Therefore, liposomal nanocarrier is a good candidate for transdermal delivery of Ce6 and CpG for tumor photoimmunotherapy. Compared with the reported nanocarriers MOFs [30], TiO_2_ nanoparticles [31], graphene quantum dots [32], MSN [33,34], and HFn [35], lipid-based delivery systems possess superior biocompatibility and high affinity for cell membranes. This high affinity enables them to readily enter the cytoplasm via endocytosis or direct membrane fusion, and subsequently release drugs through lipid-phospholipid exchange [46]. While liposomal systems have been widely explored to co-encapsulate Ce6 with immune adjuvants such as R837 [47] or R848 [48], the transdermal co-delivery of Ce6 and CpG via liposomes specifically for synergistic photo-immunotherapy remains unreported. Despite these advancements, the transcutaneous penetrative capacity of liposomes is still limited, leading to low drug bioavailability. Transdermal-enhancing peptide TD (ACSSSPSKHCG), which can temporarily open the paracellular pathway and accelerate penetration of exogenous pharmaceutical compounds through the skin [49]. In our previous work, we have successfully harnessed the TD peptide to markedly enhance the transdermal delivery efficacy of vemurafenib-loaded liposomes [50] and lipid-modified iron oxide nanocomplex [51].

**Fig. 1:**
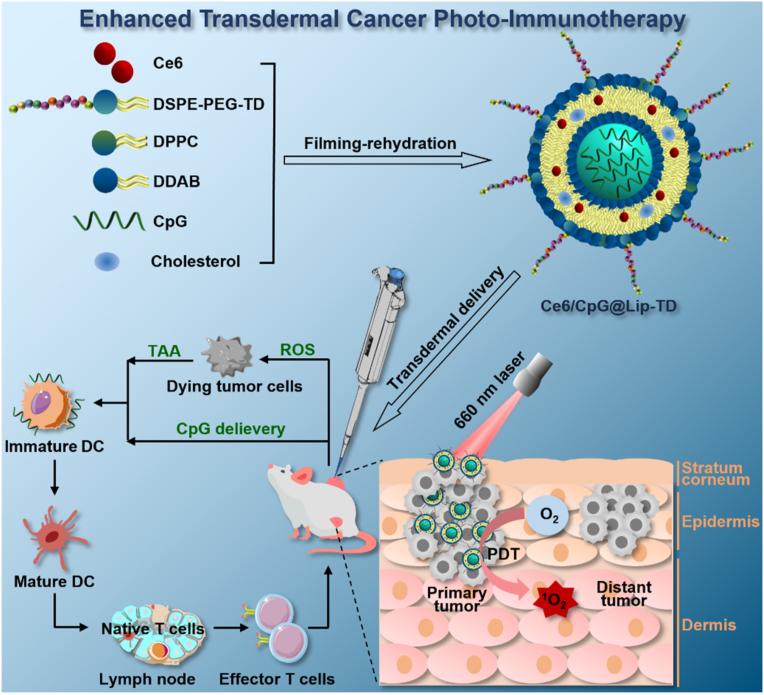
Schematic preparation of Ce6/CpG@Lip-TD and its transdermal delivery for photo-immunotherapy of superficial tumors. The TD-functionalized cationic liposome Ce6/CpG@Lip-TD penetrates the skin and accumulated in the tumor. Upon irradiation, Ce6/CpG@Lip-TD can mediate PDT to directly destroy primary tumor cells and induce ICD. ICD combined with immunoadjuvant CpG significantly promote DC maturation and subsequently initiate T-lymphocyte-mediated systemic antitumor immunity to inhibit distant tumor.

Herein, a TD-modified cationic liposome encapsulating the photosensitizer Ce6 and the immunoadjuvant CpG, denoted as Ce6/CpG@Lip-TD, was rationally designed to mediate photo-immunotherapy of superficial tumors via transdermal administration (Fig. 1). Upon topical application of Ce6/CpG@Lip-TD to the skin at the tumor site, the liposomes effectively penetrate the skin and concentrate within the tumor tissue. Upon irradiation, Ce6/CpG@Lip-TD-mediated PDT not only directly destroys primary tumor cells but also induces ICD. ICD combined with immunoadjuvant CpG significantly promote DC maturation and subsequently initiate a T-lymphocyte-mediated systemic antitumor immunity to inhibit distant tumor. There are several advantages of Ce6/CpG@Lip-TD. Primarily, the functionalization with the TD peptide significantly enhances the dermal penetration capability of the liposomal construct. Secondly, the positively charged surface of the liposomes enhances their adhesion and interaction with the skin barrier [52]. Additionally, the inclusion of the photosensitizer Ce6 is capable of not only eradicating tumor cells directly but also inducing immunogenic cell death. Furthermore, the presence of the immunoadjuvant CpG serves to stimulate DC maturation and bolstering the anti-tumor immune response. This synergistic approach potentiates the immune effects triggered by photodynamic therapy. Moreover, the transdermal administration of the biocompatible Ce6/CpG@Lip-TD largely minimizes undesirable systemic side effects, holding great potential for clinical translation.

## 2 Materials and methods

### 2.1 Preparation materials

1,2-dihexadecanoyl-rac-glycero-3-phosphocholine (DPPC) were purchased from AVT Pharmaceutical Tech Co., Ltd. (Shanghai, China). 1,2-Distearoyl-sn-glycero-3-phosphoethanolamineN-[succinimidyl(polyethylene glycol)-2000] (DSPE-PEG2000-NHS) and dimethyl dioctadecylammonium bromide (DDAB) was purchased from Xi'an Ruixi Biological Technology Co., Ltd (Xi'an, China). Chlorine 6 (Ce6) was purchased from Frontier Scientific, Inc. Transdermal enhanced peptide TD (ACSSSPSKHCG) was synthesized by Bankpeptide Biological Technology Co., Ltd. (Hefei, China). Cholesterol, CpG, 3-(4, 5-dimethylthiazol-2-yl)-2, 5-diphenyltetrazolium bromide (MTT) and dialysis bags (3.5 kD and 10 kD) were purchased from Sangon Biotech Co., Ltd. (Shanghai, China). Calcein-AM was purchased from Shanghai Aladdin Biochemical Technology Co., Ltd. (Shanghai, China). Dimethyl sulfoxide (DMSO), microfiltration membrane filters (0.22 μm) and other reagents were purchased from Sinopharm Chenmical Reagent Co.Ltd. (Shanghai, China). AlexaFluor 488-CRT antibody was purchased from Cell Signaling Tech Co., Ltd (USA). Anti-Mo MHC II-super bright 436 and Anti-Mo CD80-PE were purchased from eBioscience (USA).

### 2.2 Cells and animals

Murine melanoma cells B16F10 and mouse breast cancer cells 4T1 were cultured in DMEM medium supplemented with 10 % FBS, 100U mL^−1^ penicillin and 0.1 mg mL^−1^ streptomycin. Male SD rats (200 ± 20 g) and female BALB/c mice (20 ± 2 g) were provided by Anhui Medical University (Hefei, China). C57BL/6J mice (20 ± 2 g) were purchased from the SPF Biotechnology Co, Ltd. Beijing, China. All animal experiments were approved by the Laboratory Animal Management Committee of the University of Science and Technology of China (approval number: USTCACUC1702007).

### 2.3 Preparation of DSPE-PEG-TD

TD peptide (10 mg) and DSPE-PEG-NHS (50 mg) were mixed into DMF (3 mL), and the pH was adjusted to 8.5 using triethylamine. After stirring at room temperature for 24 h, the solution was dialyzed using a dialysis membrane with a molecular weight cutoff of 3.5 kD for 48 h. Subsequently, the post-dialysis solution was lyophilized using a vacuum freeze dryer (Ningbo Scientz Biotechnology Co., Ltd., Ningbo, China). The successful conjugation of TD with DSPE-PEG-NHS was confirmed by gel permeation chromatography detection.

### 2.4 Preparation of Ce6/CpG@Lip-TD

The liposome Ce6/CpG@Lip-TD was prepared by a filming-rehydration method [53]. Briefly, the lipid mixture of DPPC, cholesterol, DSPE-PEG-TD, DDAB and Ce6 at a mass ratio of 10: 2.5: 4: 1.5: 0.5 were dissolved in about 6 mL of mixed organic solvent of chloroform and methanol (2:1, v/v). The solution was evaporated using a rotary evaporator to yield a thin film of lipid. Afterwards, the dried lipid film was hydrated with 1 mL of CpG aqueous solution (5 μM) containing 0.05 % Tween 80 and stirred at 40 °C for 30 min followed by being sonicated for 10 min (60 W, ultrasonic for 3 s with 2 s interval).

### 2.5 Morphology

The morphological characteristics of Ce6/CpG@Lip and Ce6/CpG@Lip-TD were verified using cryogenic transmission electron microscopy (Glacios Cryo-TEM; FEI Company, Hillsboro, USA) with a 200 kV accelerating voltage.

2.6. Fluorescence property and UV Absorption.

The fluorescence spectra and UV absorbance of Ce6/CpG@Lip and Ce6/CpG@Lip-TD were recorded using a fluorescence spectro-photometer (F-7100, Hitachi High-Tech, Japan) and a NanoDrop 2000 (Thermo Scientific, USA), respectively.

Size and Zeta Potential: The size distribution and zeta potential of Ce6/CpG@Lip and Ce6/CpG@Lip-TD were measured using a dynamic light scattering system (Malvern Zetasizer-UV-Malvern Instruments Ltd, Malvern, UK).

### 2.6 Encapsulation efficiency of Ce6

To calculate the Ce6 encapsulation efficiency of liposomes, a standard curve of Ce6 was firstly established (Fig. S1). The obtained regression equation was used to determine the Ce6 concentration. Ce6 loaded liposome samples were destroyed with methanol to dissolve Ce6 and then centrifuged at 10,000 rpm for 10 min to remove the liposome remnants. The UV absorbance distributions of destroyed liposome samples were determined using a nanodrop. The UV absorbance distribution of methanol was used as a baseline. Based on this regression equation, the concentration of Ce6 in Ce6/CpG@Lip-TD can be determined. Encapsulation efficiency of Ce6 = (quantity of loaded Ce6/total quantity of Ce6) × 100 %.

### 2.7 Agarose gel electrophoresis

To verify the successful incorporation of CpG into the liposome, agarose gel electrophoresis experiments were conducted. A 3 % agarose solution was prepared by dissolving ultrapure agarose (1.5 g) in Tris-acetate-EDTA (TAE) buffer (50 mL). Ethidium bromide (EtBr) was added to the melted agarose, and the mixture was subsequently cooled to room temperature on a cast. Free CpG (5 μM) and Ce6/CpG@Lip-TD samples (5 μM CpG) combined with 6 × loading dye were loaded onto the gel. The gel was electrophoresed at 120 V for 20 min in TAE buffer and then visualized using the Gel imager system (GelX1620).

### 2.8 Evaluation of the singlet oxygen generation ability of Ce6/CpG@Lip-TD

The solutions of free Ce6, Ce6/CpG@Lip and Ce6/CpG@Lip-TD at a Ce6 concentration of 5 μM in phosphate buffered solution (PBS) were mixed with a commercial singlet oxygen sensor green (SOSG) probe at a final concentration of 2.5 μM and then subjected to a 660 nm laser at 5 mW cm^−2^. At different time points of 5, 10, 15, 20, 25 and 30 min post-irradiation, sample (100 μL) was pipetted out from each well, and the fluorescence intensity of SOSG was recorded using a multimode microreader.

### 2.9 In vitro cytotoxicity and photodynamic toxicity of Ce6/CpG@Lip-TD

The NIR light activatable cytotoxicity and dark toxicity of Ce6/CpG@Lip-TD were evaluated on both B16F10 cells and 4T1 cells by utilizing the standard MTT assay. B16F10 cells or 4T1 cells were seeded in 96-well plates (100 μL each well) at a density of 1 × 10^4^ cells mL^−1^ and cultured at 37 °C with 5 % CO_2_ for 24 h. Then, the culture medium in the 96-well plates was replaced with 100 μL of fresh medium containing free Ce6, Ce6/CpG@Lip or Ce6/CpG@Lip-TD at Ce6 equivalent concentration ranging from 0 to 10 μM. After the cells were incubated for 24 h, the medium containing materials were removed from the wells and 10 μL of 5 mg mL^−1^ MTT was added, and the plates were incubated for another 4 h. Subsequently, the MTT solution was replaced with DMSO (150 μL), and the plates were shaken constantly in the dark. The absorbance of the solution in each well of the 96-well plates at 570 nm was measured using the ELISA microplate reader. Cell viability under each condition was calculated according to the absorbance values.

To assess the photodynamic toxicity of Ce6/CpG@Lip-TD, the cells were incubated with free Ce6, Ce6/CpG@Lip, or Ce6/CpG@Lip-TD for 4 h. Following this incubation period, the culture media were carefully removed, and the cells were washed twice with PBS. Fresh medium was then added to the cells, which were subsequently exposed to a 660 nm laser at an intensity of 5 mW cm^−2^ for 10 min. After incubating for another 20 h post-laser irradiation, the cells were performed with standard MTT assay.

### 2.10 Live/dead cell staining assay

B16F10 cells were cultured with medium containing free Ce6, Ce6/CpG@Lip or Ce6/CpG@Lip-TD at various Ce6 concentrations for 4 h. Then the culture media were replaced with fresh medium and cells were subsequently exposed to a 660 nm laser at an intensity of 5 mW cm^−2^ for 10 min. After incubating for another 20 h post-laser irradiation, Calcein-AM and PI were used for live/dead cell staining for 10 min. Finally, the cell viability observed under a fluorescence microscope.

### 2.11 Cellullar uptake

B16F10 cells or 4T1 cells were seeded on a confocal dish at a density of 1 × 10^4^ per well. 24 h later, cells were incubated with free Ce6, Ce6/CpG@Lip or Ce6/CpG@Lip-TD at a Ce6 concentration of 10 μM in DMEM (200 μL) and incubated for1, 2 or 4 h respectively. Then the cells were washed with PBS twice and fixed with 4 % paraformaldehyde solution for 20 min. The nuclei were then stained and mounted with Vectashield mounting medium containing 4,6-diamidino-2-phenylindole (DAPI). The cellular uptake of each sample was assessed by confocal microscopy (ZEISS710, Germany).

### 2.12 Immunofluorescence assay of CRT in vitro

The surface-exposure of CRT was assessed by immunofluorescence. For immunofluorescence analysis, B16F10 cells were seeded into confocal dishes (1 × 10^5^ cells) and cultured for 24 h. Then the cells were treated with PBS or Ce6@Lip-TD (10 μM Ce6). After 4 h incubation, cells were irradiated with a 660 nm laser for 10 min (5 mW cm^−2^). After further 24 h incubation, the cells were washed twice with PBS and then incubated with Alexa Fluor® 488-CRT antibody and stained with DAPI for confocal imaging.

### 2.13 Evaluation of DC maturation

Bone-marrow-derived cells (BMDCs) were used to evaluate the DC maturation mediated by Ce6/CpG@Lip-TD. BMDCs were isolated, activated and differentiated into dendritic cells according to a previously published protocol [29]. In a sterile environment, the ends of each hind legs from BALB/c mice were excised and an insulin syringe filled with RPMI complete medium was inserted into the exposed bone end to flush out the bone marrow into a cell culture dish filled with RPMI complete medium. This process was repeated until the bone appeared white and translucent. The obtained bone-marrow-derived monocytic cells were further cultured with GM-CSF (20 ng mL^−1^) and IL-4 (10 ng mL^−1^) for 5 days.

B16F10 cells were seeded in 96-well plates (100 μL per well) at a density of 1 × 10^4^ cells mL^−1^ and cultured for 24 h. The culture medium in each well was then replaced with 100 μL of fresh medium containing free Ce6 or Ce6@Lip-TD at an equivalent Ce6 concentration (10 μM) for 4 h. After removing the medium and washing cells with PBS, the cells were treated with or without laser irradiation (5 mW cm^−2^, 10 min). Subsequently, immature DCs were added to each well and incubated for 24 h. DCs were then collected and stained with anti-mouse MHC II and anti-mouse CD80 antibodies, and then analyzed by flow cytometry.

### 2.14 In vitro penetration study

The transdermal capability of the Ce6/CpG@Lip-TD was investigated using the Franz diffusion cell system. A male SD rat was anaesthetized prior to being sacrificed. The hair of the abdominal skin (∼3 cm × 3 cm) was removed and then collected the skin and removed the subcutaneous fat. The skin without any defects was mounted on the Franz diffusion cell system (Tianjin Zhengtong Technology Co., Ltd., Tianjin, China) with a 5 mL receptor compartment and a 1 cm diameter diffusion area. Next, PBS (5 mL) was added to the receptor and Ce6/CpG@Lip (0.2 mL) or Ce6/CpG@Lip-TD (0.2 mL) was added to donor compartments. Then, the samples (200 μL) were taken from the receptor at time points of 4, 8, 12, and 24 h, respectively. Methanol (200 μL) was added to each sample to destruct the liposomes, the solutions were centrifuged at 10,000 rpm for 10 min to remove the liposome reminants. Finally, the concentrations of Ce6 in the solutions were determined using a nanodrop.

### 2.15 Establishment of tumor models

A bilateral subcutaneous melanoma-bearing mouse model and a bilateral subcutaneous breast tumor-bearing mouse model were established using B16F10 cells and 4T1 cells respectively. For the melanoma-bearing mouse model, the primary tumor was established via subcutaneous injection of B16F10 cells (1 × 10^6^) into the left flank of a female C57BL/6J mouse, and the distant tumor was conducted via subcutaneously injecting B16F10 cells (2 × 10^5^) into the right flank of the same mouse on day 6. For the breast tumor-bearing mouse model, same procedure was operated except for using 4T1 cells on BALB/c mice. The tumor volume was calculated in accordance with the following formula: width^2^ × length × 0.5.

### 2.16 In vivo antitumor research

The in vivo antitumor study was performed on bilateral B16F10 bearing mice. To study the anti-tumor and abscopal effect of Ce6/CpG@Lip-TD mediated therapy, the tumor-bearing mice were divided into 8 groups (n = 5): (1) PBS (L-), (2) PBS (L+), (3) Ce6/CpG@Lip (L-), (4) Ce6/CpG@Lip (L+), (5) Ce6@Lip (L-), (6) Ce6@Lip (L+), (7) Ce6/CpG@Lip-TD (L-) and (8) Ce6/CpG@Lip-TD (L+). 7 days after the primary tumor inoculation, the bilateral B16F10 bearing mice were administered via transdermal with PBS, Ce6/CpG@Lip, Ce6@Lip-TD and Ce6/CpG@Lip-TD at a Ce6 concentration of 4.65 mg kg^−1^, respectively. After 24 h, the primary tumors of mice in each group were irradiated with or without a 660 nm laser for 10 min (100 mW cm^−2^). Drug administration and irradiation treatment were performed only once. The primary and distant tumor size (tumor volume = length × width^2^/2) and body weight were monitored every two days. For the evaluation of the anti-tumor effect of Ce6/CpG@Lip-TD in 4T1 tumor models, the tumor-bearing mice were divided into 6 groups (n = 5): (1) PBS (L+), (2) CpG@Lip-TD (L+), (3) Ce6/CpG@Lip (L+), (4) Ce6@Lip-TD (L+), (5) Ce6/CpG@Lip-TD (L-) and (6) Ce6/CpG@Lip-TD (L+). The experimental procedures for these groups were identical to those used in the B16F10 tumor models.

### 2.17 Ex vivo immunofluorescence staining and flow cytometry analysis

For detection of CRT exposure, T cell infiltration and production of cytokines after therapy, the primary tumors of various groups were harvested on day 2 after treatments and incubated with the primary antibodies (anti-CRT; anti-CD8; anti-IL-6 and anti-TNF-α) and relevant fluorescein labeled secondary antibodies according to the manufacturer's protocol. After staining with DAPI, the acquired slices anchored with different fluorescein were observed under CLSM.

For analysis of the DC maturation and NK activation in vivo, the tumor draining lymph nodes (TDLNs) of each group of mice were harvested 2 days after treatments. The TDLNs were filtered with nylon mesh filters to acquire the single-cell suspension. To analyze the activated DCs, the cells were stained with anti-mouse CD11C-FITC, anti-mouse CD86-PE and anti-mouse CD80-APC antibodies. To analyze the activated NK cells, the cells were stained with NK1.1-BV605 and CD69-pc5.5 and subsequently analyzed by flow cytometry.

### 2.18 In vivo biosafety of Ce6/CpG@Lip-TD

In vivo toxicity was explored by organ functions detection from mice in PBS and Ce6/CpG@Lip-TD-treatment groups. 100 μL PBS or suspension of Ce6/CpG@Lip-TD (6.2 mg/kg Ce6) were administered via transdermal into healthy mice (n = 5). After a one-week post-administration period, blood samples were collected by orbital puncture and serums were separated by centrifugation. Subsequently, the biomarker levels, including alanine transaminase (ALT), aspartate aminotransferase (AST), alkaline phosphatase (AKP) and blood urea nitrogen (BUN) and creatinine (CREA), were tested by serum biochemical analyses using corresponding kits to reflect the liver and kidney functions. Mice treated by PBS or Ce6/CpG@Lip-TD (6.2 mg/kg Ce6) with or without laser irradiation (100 mW cm^−2^, 10 min) were sacrificed to collect main organs for histological study.

### 2.19 Statistical analysis

Data are presented as means ± SD and analyzed using a two-tailed Student's *t*-test or one-way analysis of variance (ANOVA) where appropriate, with the following significance values: ns, not significant; ∗*P* < 0.05, ∗∗*P* < 0.01, ∗∗∗*P* < 0.001, and ∗∗∗∗*P* < 0.0001. Statistical calculations were performed using Origin 2019 or GraphPad Prism v. 8.0.1.

## 3 Results

### 3.1 Preparation and characterization of Ce6/CpG@Lip-TD

**Fig. 2:**
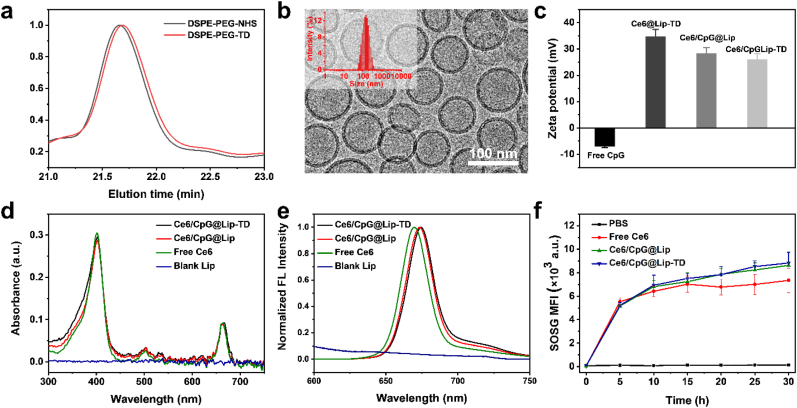
Characterization of Ce6/CpG@Lip-TD. (a) GPC profiles of DSPE-PEG-NHS and DSPE-PEG-TD. (b) Cryo-TEM image of Ce6/CpG@Lip-TD (insert: the hydrodynamic size distribution of Ce6/CpG@Lip-TD). (c) Zeta potential of free CpG, Ce6@Lip-TD, Ce6/CpG@Lip and Ce6/CpG@Lip-TD (mean ± SD, *n* = 3). (d) UV absorption spectra and (e) fluorescence spectra of free Ce6 in 12 % methanol solution, blank liposome, Ce6/CpG@Lip, and Ce6/CpG@Lip-TD in water. (f) ^1^O_2_ generation abilities of PBS and free Ce6, Ce6/CpG@Lip, Ce6/CpG@Lip-TD determined using SOSG (mean ± SD, *n* = 3 independent experiments).

The transdermal-enhancing peptide conjugated lipid DSPE-PEG-TD was initially synthesized by a facile reaction of DSPE-PEG-NHS with the amine group of TD peptide. Gel permeation chromatography (GPC) elution traces revealed a larger molecular weight of DSPE-PEG-TD compared with DSPE-PEG-NHS, indicating the successful conjugation (Fig. 2a). Matrix-assisted laser desorption ionization time of-flight mass spectrometry (MALDI-TOF-MS) analysis of TD, DSPE-PEG-NHS and DSPE-PEG-TD further confirmed successful attachment of TD to DSPE-PEG-NHS (Fig. S2). Ce6/CpG@Lip-TD was prepared by a filming-rehydration method [53]. A liposome Ce6/CpG@Lip without TD and a liposome Ce6@Lip-TD without CpG were prepared using the same procedure to serve as control groups. Cryogenic transmission electron microscopy (cryo-TEM) images showed that the obtained Ce6/CpG@Lip-TD and Ce6/CpG@Lip possess a regular spherical morphology with a smooth lipid surface (Fig. 2b and S3). Dynamic light scattering (DLS) measurement revealed the average size of Ce6/CpG@Lip-TD to be 125 nm (Fig. 2b and Table S1). Ce6/CpG@Lip showed an average size of 121 (Fig. S4 and Table S1), which was similar to that of Ce6/CpG@Lip-TD. Ce6/CpG@Lip-TD exhibited a positive surface charge with a zeta potential of 26.1 ± 2.04 mV due to the presence of a small amount of cationic lipid DDAB within the liposomal formulation (Fig. 2c and Table S1), which can facilitate skin permeation and cellular uptake [54]. Ce6/CpG@Lip exhibited a similar zeta potential (28.3 ± 2.22 mV) to that of Ce6/CpG@Lip-TD, indicating minimal alteration in liposomal surface charge following TD modification. Both Ce6/CpG@Lip-TD and Ce6/CpG@Lip displayed a slight lower zeta potential compared to Ce6@Lip-TD (34.7 ± 2.72 mV), which might be due to a small amount of negatively charged CpG adsorption on the surface of the cationic liposomes [55]. Agarose gel electrophoresis experiments also verified the successful incorporation of CpG into the liposomes and the CpG encapsulation efficiency was measured as about 92 % (Fig. S5). To verify that Ce6 was successfully encapsulated into liposomes, the UV absorbance and fluorescence spectra of blank liposome, free Ce6, Ce6/CpG@Lip, and Ce6/CpG@Lip-TD were measured. As expected, Ce6/CpG@Lip-TD and Ce6/CpG@Lip showed characteristic peaks of Ce6 at 402 nm and 663 nm on its UV/Vis-NIR absorbance spectrum (Fig. 2d), indicating successful encapsulation. Fluorescence spectra of both Ce6/CpG@Lip-TD and Ce6/CpG@Lip revealed a distinctive emission peak of Ce6 at 675 nm, further confirming successful encapsulation of Ce6 (Fig. 2e). The Ce6 encapsulation efficiencies of Ce6/CpG@Lip-TD and Ce6/CpG@Lip were measured as high as 94.3 ± 2.87 % and 93.7 ± 2.16 %, respectively. Moreover, the Ce6 encapsulation efficiencies remained nearly unchanged over a 7-day period, indicating the high stability of Ce6/CpG@Lip-TD and Ce6/CpG@Lip (Fig. S6).

To investigate the potential influence of liposomal encapsulation on the photodynamic property of Ce6, the ^1^O_2_ generation capacity of Ce6/CpG@Lip-TD and Ce6/CpG@Lip was assessed using the singlet oxygen sensor green (SOSG) probe. Remarkably, both Ce6/CpG@Lip-TD and Ce6/CpG@Lip exhibited robust ^1^O_2_ generation after exposure to a 660 nm laser, similar to the performance observed with free Ce6 (Fig. 2f). This result demonstrated the effectiveness of Ce6/CpG@Lip-TD as a nanophotosensitizer.

### 3.2 In vitro cytotoxicity of Ce6/CpG@Lip-TD

**Fig. 3:**
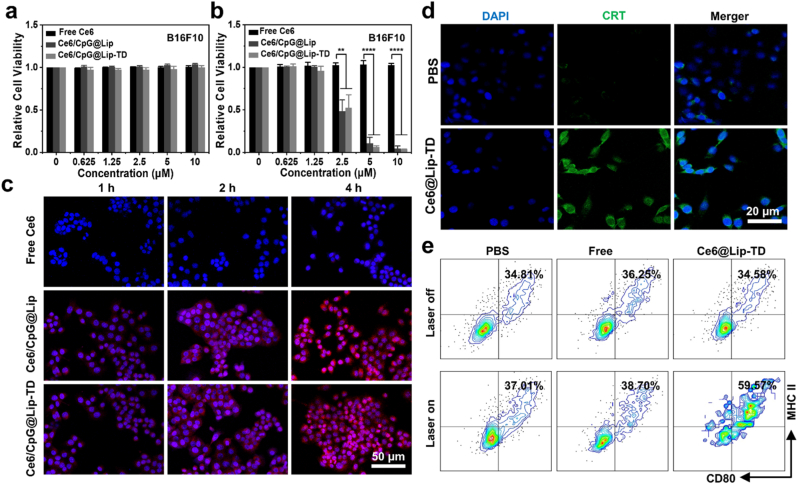
Cytotoxicity and cellular uptake of Ce6/CpG@Lip-TD and in vitro activation of immune response elicited by PDT. (a,b) Relative viabilities of B16F10 cells incubated with free Ce6, Ce6/CpG@Lip and Ce6/CpG@Lip-TD for 4 h, then irradiated **(a)** without or **(b)** with a 660 nm laser for 10 min followed by additional 20 h incubation before the standard MTT assay (mean ± SD, data were based on three independent experiments, analyzed by a two-tailed unpaired Student's *t*-test, ∗∗*P* < 0.01, ∗∗∗∗*P* < 0.0001). (c) Confocal images of intracellular internalization profiles of free Ce6, Ce6/CpG@Lip and Ce6/CpG@Lip-TD after incubation with B16F10 cells for different time. DAPI (blue) and Ce6 (red) were excited at 404 nm and 488 nm respectively. (d) Confocal images of CRT (green) expression on the cell surface of B16F10 cells treated with PBS and Ce6@Lip-TD with light irradiation, with nuclei co-stained with DAPI (blue). (e) Flow cytometry assessment of DC maturation via the analysis of two key biomarkers (MHC II and CD80).

Preceding any biomedical application, the cytotoxicity of Ce6/CpG@Lip-TD underwent careful evaluation via Methylthiazolyldiphenyl-tetrazolium bromide (MTT) assay using B16F10 murine melanoma cells and 4T1 mouse mammary tumor cells. Impressively, Ce6/CpG@Lip-TD exhibited negligible cytotoxic effects on both B16F10 and 4T1 cells, even at Ce6 dosages as high as 10 μM, demonstrating its excellent biocompatibility (Fig. 3a and S7a). Subsequently, to evaluate the potential photodynamic cytotoxicity of Ce6/CpG@Lip-TD against cancer cells, cells were exposed to a 660 nm laser (5 mW cm^−2^, 10 min). Upon laser irradiation, the cell viabilities of both B16F10 and 4T1 decreased significantly as the Ce6 concentrations of Ce6/CpG@Lip and Ce6/CpG@Lip-TD increased above 2.5 μM, culminating in near-total cellular eradication at a Ce6 concentration of 10 μM (Fig. 3b and S7b). To accurately evaluate photodynamic cytotoxicity of Ce6/CpG@Lip-TD, we further conducted additional experiments using Ce6 concentrations ranging from 1.25 to 2.5 μM in both cell models. The results showed that phototoxicity of Ce6/CpG@Lip-TD upon laser irradiation increased progressively with increasing concentration (Fig. S8). In sharp contrast, free Ce6 evinced minimal cytotoxicity even at the highest Ce6 concentrations under identical irradiation conditions. We conjecture that this phenomenon stems from the inherently low cellular uptake of free Ce6, whereas the liposomal encapsulation of Ce6/CpG@Lip and Ce6/CpG@Lip-TD could enhance their cellular internalization, thereby resulting in a potent cell-killing effect upon exposure to laser irradiation.

Furthermore, to observe the therapeutic effect visually, we carried out cell co-staining using calcein-AM (staining live cells in green) and PI (staining dead cells in red). Upon irradiation, the proportion of cell death increased gradually as the concentration of Ce6/CpG@Lip or Ce6/CpG@Lip-TD increased (Fig. S9). Notably, at a Ce6 concentration of 10 μM, the free Ce6-treated group displayed bright green fluorescence, whereas nearly all cells treated with Ce6/CpG@Lip and Ce6/CpG@Lip-TD exhibited conspicuous red fluorescence. These results are consistent with the aforementioned MTT findings. Taken together, the biocompatible Ce6/CpG@Lip-TD demonstrated potent photodynamic cytotoxicity, making it a promising candidate for photodynamic therapy-driven cancer management.

### 3.3 Intracellular uptake of Ce6/CpG@Lip-TD

To assess the intracellular uptake of Ce6/CpG@Lip-TD, we conducted confocal laser scanning microscopy (CLSM) analysis on both B16 and 4T1 cancer cells incubated with free Ce6, Ce6/CpG@Lip, or Ce6/CpG@Lip-TD for varying durations. The results showed that the intensity of red fluorescence originating from Ce6 increased over time in both the Ce6/CpG@Lip and Ce6/CpG@Lip-TD treatment groups, indicating a time-dependent cellular uptake behavior (Fig. 3c and S10). In contrast, cells treated with free Ce6 exhibited minimal red fluorescence. Notably, significant intracellular internalization was observed within 1 h of incubation with Ce6/CpG@Lip and Ce6/CpG@Lip-TD, whereas minimal internalization was found in cells treated with free Ce6 even after 4 h. These findings suggest that free Ce6 has limited ability to penetrate the cancer cell membrane, while the cationic liposomal carriers containing Ce6 facilitate efficient cellular internalization. Compared to other nanocarriers co-loaded with CpG and Ce6, such as MOFs [30], TiO_2_ nanoparticle [31], graphene quantum dots [32], MSN [33,34] and HFn [35], our liposomes exhibited significantly higher or comparable cellular uptake efficiency, which is attributed to the high affinity of liposomes for cell membranes, facilitating more effective drug delivery. This phenomenon also elucidated the photodynamic toxicity outcomes delineated in Fig. 3b. The efficacious cellular uptake of Ce6/CpG@Lip and Ce6/CpG@Lip-TD can be attributed to two aspects: On one hand, the amphiphilic properties of phospholipids within liposomes mimics the constitution of natural cell membranes, thus facilitating interactions between liposomes and cancer cell membranes, consequently promoting highly efficacious cellular internalization. On the other hand, the positive charged surface of liposome augments their affinity for the negatively charged glycocalyx on the cell surface of cancer cells via electrostatic attraction [56]. In addition, our results indicated that the modification with the TD peptide exerted negligible influence on the cellular uptake of liposomes, possibly attributable to its negligible impact on liposome size and zeta-potential.

### 3.4 In vitro immunogenic cell death triggered by PDT

Previous studies have provided evidence that PDT has the potential to induce ICD in tumor cells, resulting in the release of immunogenic signals such as DAMPs and tumor associated antigens [19]. Among the crucial DAMPs, calreticulin (CRT) plays a vital role in stimulating the engulfment of dying tumor cells by immature DCs and subsequently promoting DC maturation [57,58]. To ascertain whether our Ce6 encapsulated liposome-mediated PDT can trigger ICD, B16F10 cells were pre-treated with Ce6@Lip-TD, followed by laser irradiation (5 mW cm^−2^, 10 min). Six hours later, the expression level of CRT was evaluated by the immunofluorescence staining using Alexa Fluor 488 (AF488)-labeled CRT antibody. Strong green fluorescence was observed in the Ce6@Lip-TD-treated groups, whereas PBS-treated groups exhibited negligible green fluorescence (Fig. 3d), indicating pronounced cell surface CRT exposure induced by Ce6 encapsulated liposome-mediated PDT.

To assess the efficacy of liposome-mediated PDT in stimulating anti-cancer immunity, we initially examined the activation of bone marrow-derived dendritic cells (BMDCs) using flow cytometry. B16F10 cells were pre-treated with Ce6@Lip-TD for 4 h, followed by laser irradiation. Subsequently, immature DCs were then introduced and co-cultured with B16F10 cells. To assess the maturation of DCs, flow cytometry analysis was performed to quantify the expression levels of surface receptors MHC II and CD80. After laser irradiation, Ce6@Lip-TD-treated group exhibited a population of matured DCs (MHC II^+^CD80^+^ DCs) constituting 59.57 % of the total co-cultured DCs, which was 1.7-fold higher than that of Ce6@Lip-TD-treated group (34.58 %) without laser irradiation (Fig. 3e). Furthermore, Ce6@Lip-TD prompted a 1.5-fold elevation in matured DC levels relative to the free Ce6-treated group following irradiation. These findings indicated that PDT mediated by Ce6-loaded liposome could induce cancer cells to release a high level of tumor-related antigens and DAMPs, thereby promoting the maturation of BMDCs.

### 3.5 Skin permeation of Ce6/CpG@Lip-TD in vitro

**Fig. 4:**
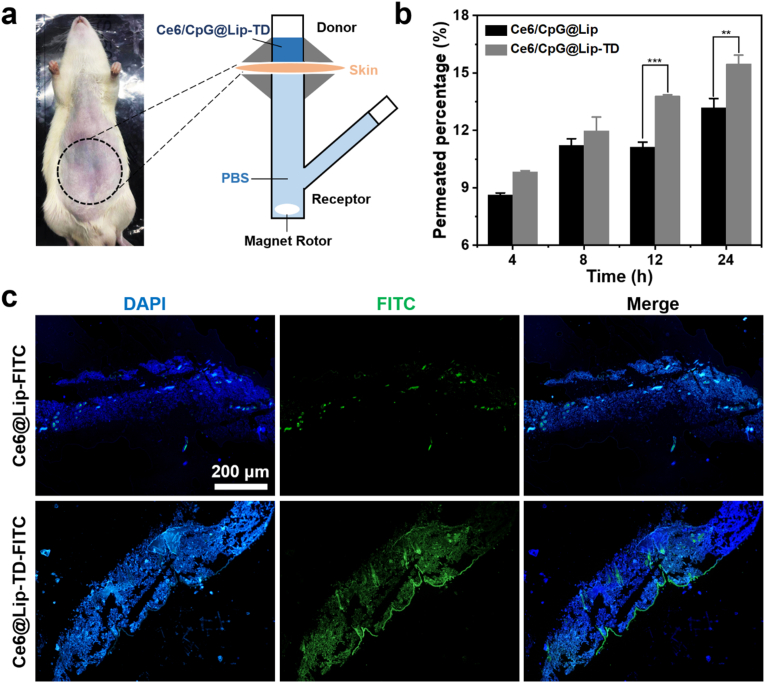
Skin permeation of Ce6/CpG@Lip-TD in vitro. (a) Schematic of the Franz diffusion cell used for in vitro permeation studies. (b) Quantification of the penetrated Ce6/CpG@Lip and Ce6/CpG@Lip-TD over time (mean ± SD, *n* = 3, analyzed by a two-tailed unpaired Student's *t*-test, ∗∗*P* < 0.01, ∗∗∗*P* < 0.001). (c) Fluorescence images of mouse skin tissues after topical treatment with Ce6@Lip-FITC (green) and Ce6@Lip-TD-FITC (green), with nuclei counterstained with DAPI (blue). Shown are representative images from three independent experiments.

To investigate whether the peptide TD could facilitate the transdermal delivery of Ce6/CpG@Lip-TD, Franz diffusion system was used to evaluate the percutaneous permeability of Ce6/CpG@Lip-TD in vitro (Fig. 4a). By quantifying the amount of Ce6 in the acceptor at various time points, we observed a gradual penetration of both Ce6/CpG@Lip and Ce6/CpG@Lip-TD through the skin. Notably, the penetration efficacy of the latter was significantly higher than that of the former after 12 h (Fig. 4b), indicating the substantial enhancement in skin permeability facilitated by the conjugated TD peptide. The progressive penetration of Ce6/CpG@Lip in the absence of TD was attributed to the favorable adsorption of cationic liposomes onto the negatively charged skin surface, followed by their subsequent penetration into through skin. To directly observe the skin penetration of TD modified liposome. FITC labeled Ce6@Lip-TD-FITC and Ce6@Lip-FITC were prepared. After 24 h treatment on Franz diffusion system, skin slices were prepared and observed by fluorescence microscopy. Skin tissues treated with Ce6@Lip-TD-FITC and Ce6@Lip-FITC both exhibited green fluorescence as depicted in Fig. 4c. Remarkably, the green fluorescence intensity observed in the Ce6@Lip-TD-FITC treated group was considerably stronger than that of the Ce6@Lip-FITC treated group, further confirming the skin penetration enhancement conferred by TD. These results demonstrated that the Ce6/CpG@Lip-TD, with TD functionalization and its cationic surface, exhibited elevated percutaneous permeability. Collectively, these findings underscored that Ce6/CpG@Lip-TD, with TD functionalization and its cationic surface, displayed heightened percutaneous permeability, showcasing the potential for transdermal drug delivery in vivo.

### 3.6 Antitumor evaluation of Ce6/CpG@Lip-TD in the bilateral tumor models

**Fig. 5:**
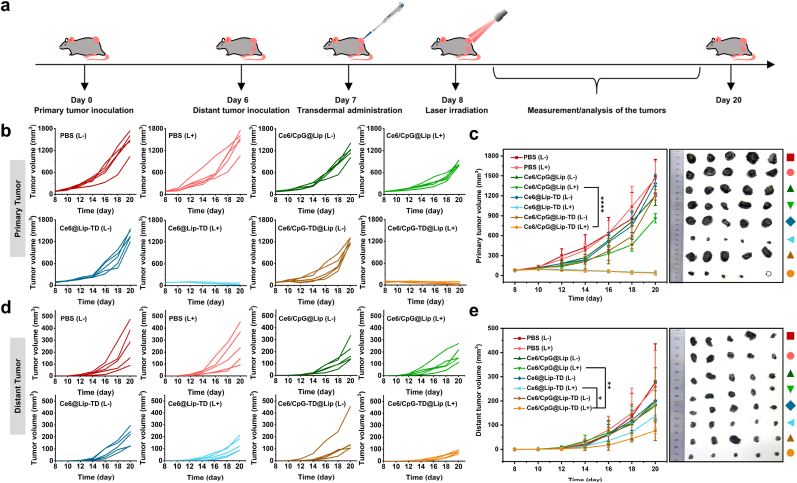
In vivo photo-immunotherapy. (a) The schematic outline shows the in vivo experimental design using B16F10 tumor-bearing mice. (b) Individual B16F10 tumor growth curves of the primary tumors receiving various treatments. (c) Time-dependent growth curves and photographs of primary B16F10 tumors. (d) Individual B16F10 tumor growth curves of the distant tumors receiving various treatments. (e) Time-dependent growth curves and photographs of distant B16F10 tumors. (*n* = 5 biologically independent animals, mean ± SD, analyzed by a two-tailed unpaired Student's *t*-test, ∗*P* < 0.05; ∗∗*P* < 0.01; ∗∗∗*P* < 0.001, ∗∗∗∗*P* < 0.0001).

The therapeutic efficacy of Ce6/CpG@Lip-TD-mediated photo-immunotherapy was assessed using a bilateral B16F10 tumor-bearing C57BL/6J mouse model. To establish the tumor model, B16F10 cancer cells were initially inoculated subcutaneously in the left flank of each mice as the primary tumor. Six days later, B16F10 cancer cells were inoculated subcutaneously in the right flank of the same mouse as the distant tumor (Fig. 5a). On day 7, primary tumor therapy was conducted by in situ transdermal administration of PBS or different liposome samples. 24 h later, the primary tumors were irradiated with or without 660 nm laser at 100 mW cm^−2^ for a duration of 10 min, whereas the distant tumors were shielded from any treatments. Note that the laser power density used here was far lower than that used in most other analogous works (Table S2). The tumor inhibition efficiency of different treatments was accessed by measuring the growth rate of the tumors every other day until the mice were sacrificed on day 20. Compared with Ce6/CpG@Lip (L+) group, Ce6/CpG@Lip-TD (L+) group displayed significant tumor inhibition efficiency (Fig. 5b and c). This result strongly demonstrated that the enhanced transdermal delivery efficiency provided by TD obviously improved the anti-tumor efficiency of phototherapy. Interestingly, we found that Ce6@Lip-TD (L+) treatment group, similar to Ce6/CpG@Lip-TD (L+) group, also elicited a robust therapeutic efficacy in combating the primary tumors progression. We think that this phenomenon was due to the enhanced penetration of Ce6 by TD, which in situ generated sufficient ^1^O_2_ to ablate tumor cells upon laser irradiation. Furthermore, in the case of the distant tumors, the therapeutic efficiency of Ce6/CpG@Lip (L+) or Ce6@Lip-TD (L+) treatment alone was significantly lower than that of Ce6/CpG@Lip-TD (L+) treatment, demonstrating a combined anti-cancer effect mediated by Ce6/CpG@Lip-TD arising from PDT provided by Ce6 and augmented immunostimulatory effects of CpG (Fig. 5d and e). In addition, no obvious fluctuations were monitored in body weight among the treated mice (Fig. S11), suggesting high therapeutic safety of the treatment. At the end of the in vivo antitumor experiment, histological examinations were conducted on the major organs (heart, liver, spleen, lung, and kidney) from all mice in each group. No significant pathological changes were detected in these organs compared to those from the PBS (L-) control group (Fig. S12), further confirming the excellent therapeutic safety.

Breast cancer carcinoma, as the most commonly diagnosed cancer among women, is the second leading cause of cancer-related mortality for women globally [59]. For this superficial malignant tumor, several studies have explored transdermal drug delivery to mitigate systemic toxicity and enhance therapeutic effectiveness [60,61]. In our previous work, we have successfully eliminated breast tumors through a transdermal approach using the widely accepted 4T1 tumor-bearing mouse model [51,62]. In this study, we also evaluated the therapeutic efficacy of Ce6/CpG@Lip-TD-mediated photo-immunotherapy on a bilateral 4T1 tumor-bearing mouse model. In line with outcomes observed in experiments on melanoma mouse model, Ce6/CpG@Lip-TD (L+) group also displayed the most significant tumor inhibition efficiency among various groups (Figs. S13–15).

### 3.7 Therapeutic mechanism

To explore the mechanism of photo-immunotherapy mediated by Ce6/CpG@Lip-TD, we next investigated PDT-induced ICD, maturation of DCs, NK cells activation, T cell infiltration, and production of cytokines using the B16F10 tumor-bearing C57BL/6J mouse model.

**Fig. 6:**
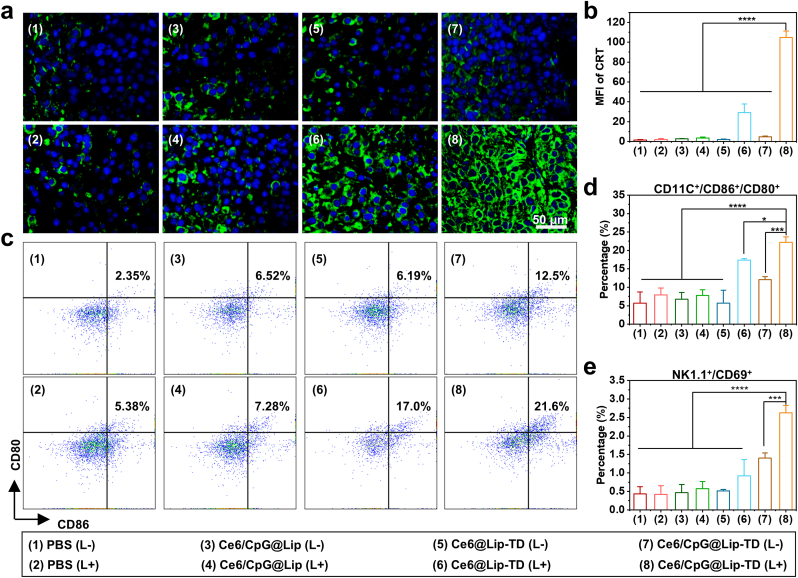
In vivo activation of immune response elicited by immunogenic PDT. (a) Immunofluorescence imaging of CRT (the typical hallmark of ICD) exposure in tumor tissues. (b) Corresponding CRT quantification in (a). (c) Flow cytometric analysis of matured DCs (CD11C^+^CD86^+^CD80^+^) in TDLNs. (d) Corresponding matured DCs quantification in (c). (e) Flow cytometric analysis of matured NK cells (NK1.1^+^CD69^+^). (*n* = 3 biologically independent animals, mean ± SD, analyzed by one-way ANOVA, ∗*P* < 0.05; ∗∗∗*P* < 0.001, ∗∗∗∗*P* < 0.0001).

CRT exposure can promote DC maturation and subsequently trigger T-lymphocyte mediated anti-tumor immunity, which is an important indicator of ICD [57,63]. We have confirmed that Ce6 mediated PDT can induce CRT exposure and DC maturation in vitro. Inspired by this, we also investigated whether the synergistic augmentation of PDT with an immunologic adjuvant could evoke robust ICD in vivo by analyzing CRT exposure using immunofluorescence staining. As shown in Fig. 6a and b, the Ce6/CpG@Lip-TD treatment group exhibited minimal green fluorescence in the absence of laser irradiation, whereas upon exposure to laser, the cells displayed a pronounced green fluorescence. Moreover, upon laser irradiation, both the Ce6@Lip-TD and Ce6/CpG@Lip-TD treatment groups exhibited bright green fluorescence compared with that of other groups. Notably, the green fluorescence intensity of the Ce6/CpG@Lip-TD treatment group was markedly higher than that of the Ce6@Lip-TD group. These findings indicated that PDT alone could induce ICD in tumor cells to a certain degree and the presence of CpG could substantially enhance this process. Additionally, in comparison to the Ce6/CpG@Lip treatment group, the Ce6/CpG@Lip-TD treatment group exhibited significantly increased CRT exposure under laser irradiation, which could be attributed to the enhanced penetration of Ce6/CpG@Lip-TD facilitated by TD.

As crucial peripheral lymphoid organs, tumor-draining lymph nodes (TDLNs) serve as pivotal sites where DCs present antigens to T cells, thereby initiating a systemic immune response [64,65]. Therefore, we collected the TDLNs of each group and analyzed their DC activation by flow cytometry. Compared with that of Ce6/CpG@Lip-TD group without laser irradiation, the abundance of mature DCs in TDLNs revealed a marked increase in the Ce6/CpG@Lip-TD group with laser irradiation, suggesting that PDT effectively induces DC maturation, thereby eliciting an immune response in vivo. Moreover, Compared with the Ce6@Lip-TD group with laser irradiation, the Ce6/CpG@Lip-TD group displayed significant higher activation level of DC cells, indicating that CpG enhances DC maturation (Fig. 6c and d), which is consistent with prior research [66]. Additionally, the Ce6/CpG@Lip-TD group with laser irradiation showed a marked increase in the proportion of activated natural killer (NK) cells in the TDLNs, compared to the Ce6/CpG@Lip-TD group without laser irradiation and Ce6@Lip-TD group with laser irradiation (Fig. 6e). These results collectively demonstrate that the combination of PDT with CpG could enhance the expression of co-stimulatory molecules on the surface of DCs, thereby promoting their maturation and consequent activation of NK cells.

**Fig. 7:**
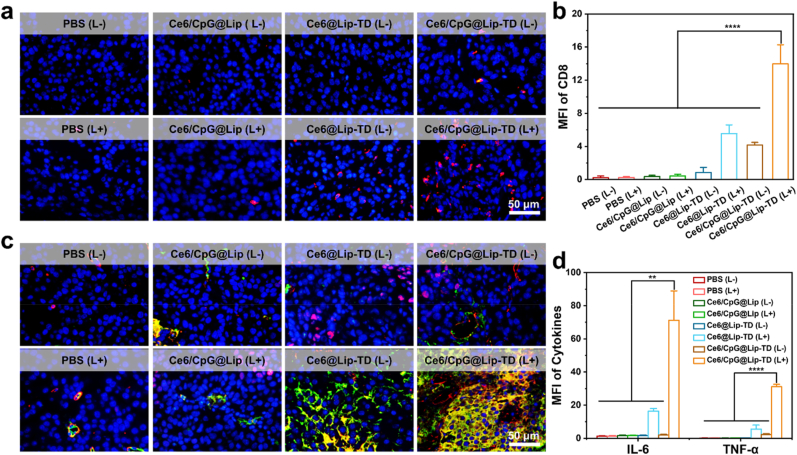
Immune cell infiltration and expression of immunosuppressive cytokines in tumor tissues. (a) Representative immunofluorescence staining detection of CD8^+^ T cells (red) in tumor tissues. (b) Corresponding CD8^+^ T cells quantification in (a). (c) Representative immunofluorescence images of TNF-α (red) and IL-6 (green) in tumor tissues, with nuclei counterstained with DAPI (blue). (d) Corresponding quantification TNF-α and IL-6 in (c). (*n* = 3 biologically independent animals, means ± SD, analyzed by one-way ANOVA,∗∗*P* < 0.01; ∗∗∗∗*P* < 0.0001).

Furthermore, to evaluate the effector T cell infiltration, the abundance of cytotoxic T lymphocytes in tumor tissues after treatment was investigated. The treatment of Ce6/CpG@Lip-TD with laser irradiation significantly promoted the infiltration of CD8^+^ T cells (red fluorescence) in the primary tumor compared with other treatments (Fig. 7a and b), indicating the activation of immune responses in vivo [67].

Subsequently, an examination of cytokine secretion was conducted to assess the immune response via immunofluorescence staining of tumor sections [58]. Tumor sections from mice subjected to Ce6@Lip-TD and Ce6/CpG@Lip-TD with laser treatments exhibited markedly elevated levels of tumor necrosis factor α (TNF-α, red fluorescence) and interleukin 6 (IL-6, green fluorescence) compared to other groups (Fig. 7c and d). Notably, the Ce6/CpG@Lip-TD treated group demonstrated even higher levels of cytokine secretion than the Ce6@Lip-TD treated group, attributed to the synergistic effects of photodynamic-induced ICD and CpG stimulation. All these results confirmed the high potency of Ce6/CpG@Lip-TD-mediated synergistic anti-cancer immunotherapy.

### 3.8 In vivo biosafety evaluation of Ce6/CpG@Lip-TD

**Fig. 8:**
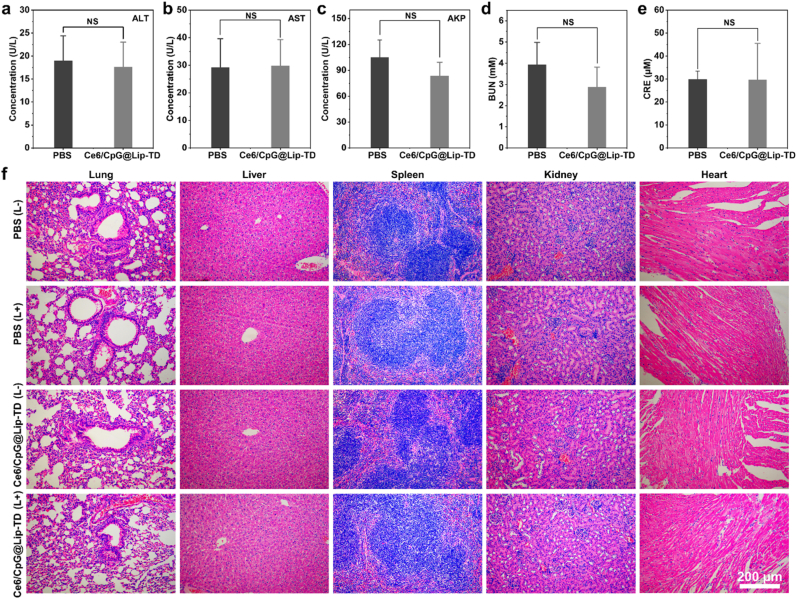
In vivo biosafety assessment. (a–c) Serum analysis of liver function with biomarker indicators of (a) ALT (b) AST and (c) AKP. (d–e) Serum analysis of kidney function with biomarker indicators of (d) BUN and (e) CRE. (f) Representative H&E-stained histological sections of organs. (*n* = 5 biologically independent animals, mean ± SD, analyzed by a two-tailed unpaired Student's *t*-test, NS (not significance)).

Ensuring biosafety is paramount for the medical application and clinical translation of any therapeutic agent. We conducted a comprehensive evaluation of the biocompatibility of Ce6/CpG@Lip-TD through serum biochemical analyses and hematoxylin and eosin (H&E) staining after transdermal application of liposomes onto the skin of healthy mice for one week. Serum biochemical analyses were performed to assess the in vivo toxicity of Ce6/CpG@Lip-TD. We measured serum levels of alanine aminotransferase (ALT), aspartate aminotransferase (AST) and alkaline phosphatase (AKP) to evaluate the liver function, as well as blood urea nitrogen (BUN) and creatinine (CREA) to evaluate the kidney function. The results, as shown in Fig. 8a–e, revealed no significant differences in these biomarkers between the treatment and control groups. In terms of histocompatibility, as shown in Fig. 8f, the organs of mice treated with Ce6/CpG@Lip-TD maintained their normal physiological morphology and exhibited no significant lesions compared to the PBS (L-) control group. Collectively, our studies did not detect any obvious signs of tissue damage or abnormal liver and kidney functions following the transdermal administration of Ce6/CpG@Lip-TD, demonstrating the high biosafety of Ce6/CpG@Lip-TD.

## 4 Conclusion

In summary, we have successfully developed a transdermal peptide-modified cationic liposome encapsulated with Ce6 and immunoadjuvant CpG for the photo-immunotherapy of superficial tumors via transdermal administration. Our findings underscored the photodynamic lethality, efficacy in promoting dendritic cell maturation in in vitro studies. In vitro and ex vivo studies demonstrated efficient transdermal permeation capability of Ce6/CpG@Lip-TD with the facilitations of it cationic surface and TD functionalization. Subsequently, in vivo assessments demonstrated substantial tumor-suppressing properties of Ce6/CpG@Lip-TD in both melanoma and breast cancer murine models, accompanied by augmented systemic immune response against distal tumors. To the best of our knowledge, the TD functionalized liposomal platform co-encapsulated with Ce6 and CpG used for photo-immunotherapy has not been reported previously. Through facile transdermal drug delivery, the Ce6/CpG@Lip-TD may provide a promising therapeutic platform for superficial cancer therapy. Importantly, the components of Ce6, CpG, liposomes, and TD peptide in the Ce6/CpG@Lip-TD system all possess high biocompatibility. Moreover, as a topical therapeutic agent, Ce6/CpG@Lip-TD circumvents the regulatory hurdles associated with systemic administration of nanomaterials and can largely decrease systemic toxicity, offering great potential for rapid translation into clinical practice.

## CRediT authorship contribution statement

**Shaozhen Wang:** Writing – original draft, Visualization, Validation, Methodology, Investigation, Formal analysis, Data curation, Conceptualization. **Chen Yang:** Methodology. **Yuanyuan Zhang:** Methodology. **Yi Hu:** Methodology. **Lan Xiao:** Methodology. **Weiping Ding:** Supervision, Resources, Project administration. **Bensheng Qiu:** Supervision, Software, Resources, Project administration, Funding acquisition, Conceptualization. **Fenfen Li:** Writing – review & editing, Writing – original draft, Supervision, Project administration, Data curation, Conceptualization.

## Declaration of competing interest

The authors declare the following financial interests/personal relationships which may be considered as potential competing interests: S.W., W.D., B.Q. and F.L. has patent licensed to ZL 2023 1 0412277.4. If there are other authors, they declare that they have no known competing financial interests or personal relationships that could have appeared to influence the work reported in this paper.

## Data Availability

Data will be made available on request.
