# Supplementary material for: Co-encapsulated Ce6 + CpG and biopeptide-modified liposomes for enhanced transdermal photo-immunotherapy of superficial tumors
Source: Mater Today Bio. 2025 Mar 13;32:101669. doi: 10.1016/j.mtbio.2025.101669 (PMC11964550; doi:10.1016/j.mtbio.2025.101669)
Supplement: Multimedia component 1 [file mmc1.pdf]

Supporting Information

Co-encapsulated Ce6 + CpG and biopeptide-modified liposomes for enhanced transdermal photo-immunotherapy of superficial tumors

Shaozhen Wang [a], Chen Yang [a], Yuanyuan Zhang [b,c], Yi Hu d, Lan Xiao [e], Weiping Ding [a,f,], Bensheng Qiu [a,]* and Fenfen Li [a,]**

[a] Medical Imaging Center, Department of Electronic Engineering and Information Science, University of Science and Technology of China, Hefei, Anhui 230026, China

[b] School of Biomedical Engineering, Division of Life Sciences and Medicine, University of Science and Technology of China, Hefei, Anhui 230026, China

[c] Suzhou Institute for Advanced Research, University of Science and Technology of China, Suzhou 215123, China

[d] Department of Burns, The First Affiliated Hospital of Anhui Medical University, Hefei, Anhui 230027, China.

[e] Department of Gynecology Oncology, The First Affiliated Hospital of USTC, Division of Life Sciences and Medicine, University of Science and Technology of China, Hefei, Anhui230031, China

[f] Department of Oncology, The First Affiliated Hospital of USTC, Division of Life Sciences and Medicine, University of Science and Technology of China, Hefei, Anhui230001, China.

Supplementary Contents:

1. Supplementary Figures (S1-S12)

2. Supplementary Tables (S1-S2)

1. Supplementary Figures (S1-S15)

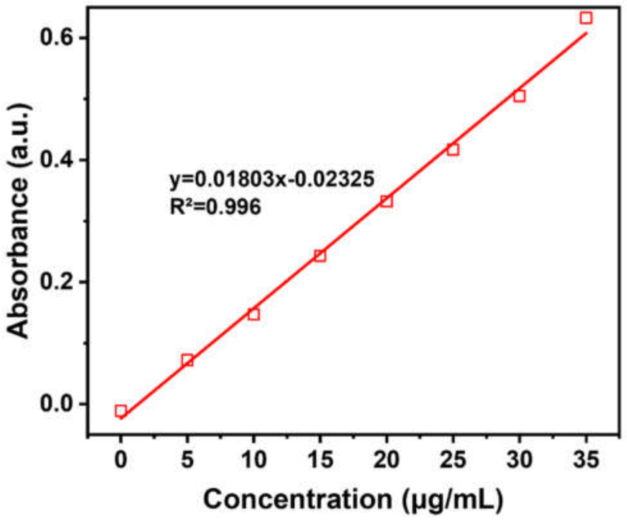

Supplementary Fig.1. The relationship between Ce6 concentration and its UV absorption value and the corresponding linear fitting curve.

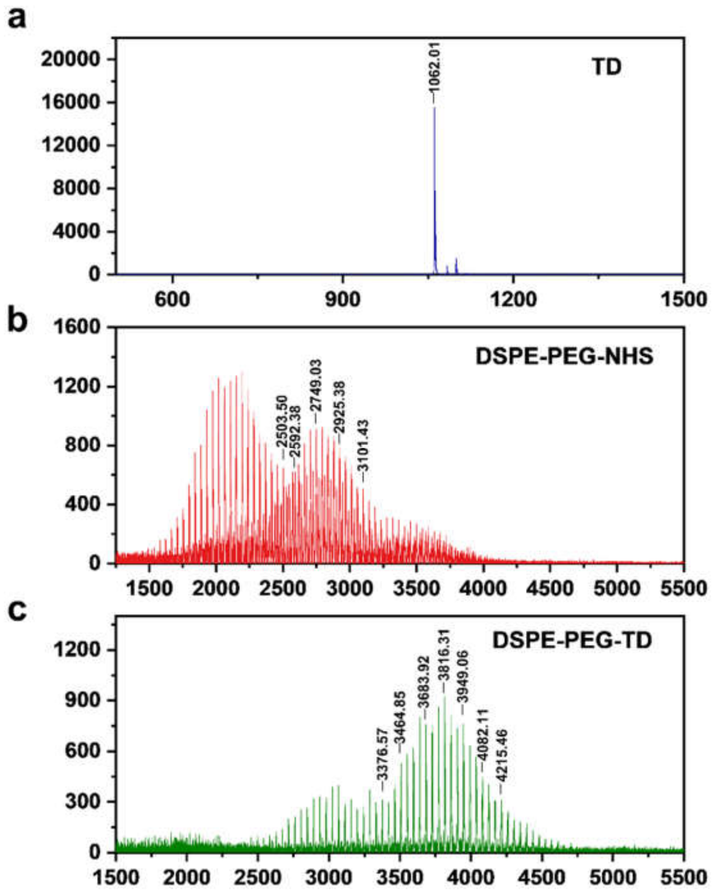

Supplementary Fig.2. MALDI-TOF-MS spectrum of DSPE-PEG-TD.

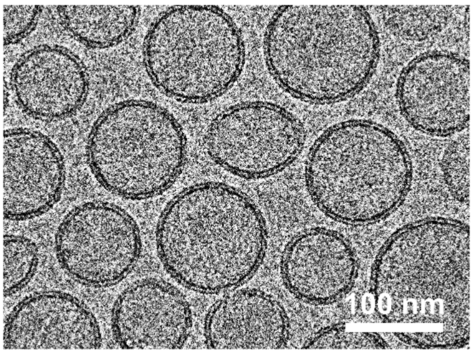

Supplementary Fig.3. Cryo-TEM image of Ce6/CpG@Lip.

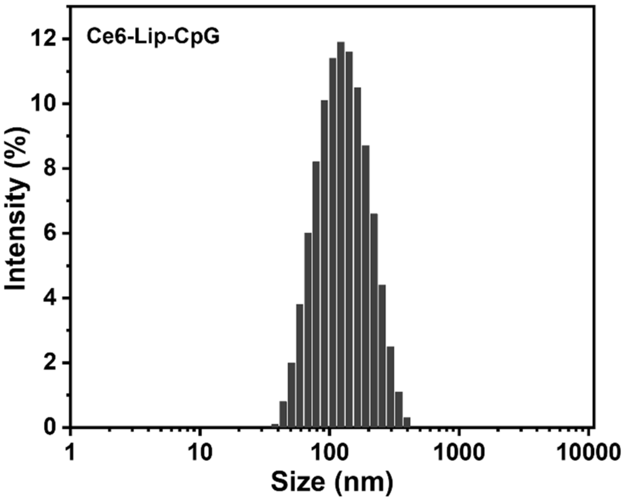

Supplementary Fig.4. The hydrodynamic size distribution of Ce6/CpG@Lip**.**

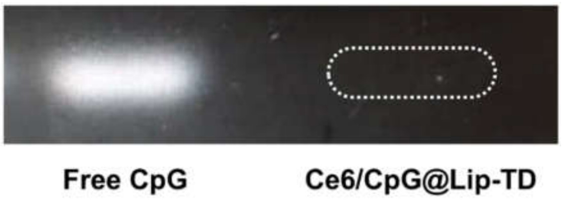

Supplementary Fig.5. Agarose gel electrophoresis images of free CpG and Ce6/CpG@Lip-TD at equivalent CpG concentrations.

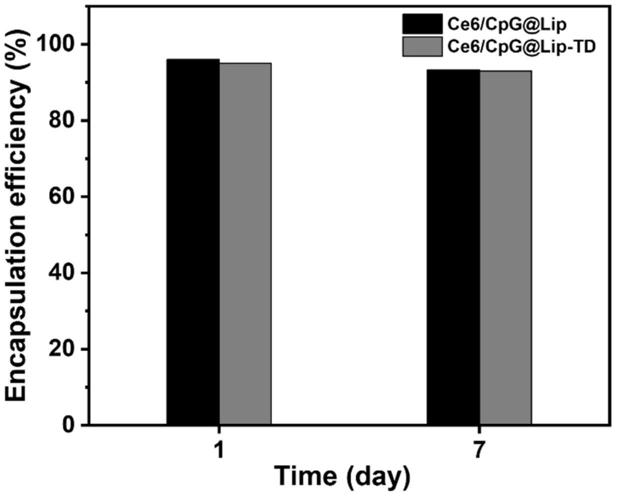

Supplementary Fig.6. Stability of Ce6/CpG@Lip. Changes of the encapsulation efficiency of Ce6/CpG@Lip and Ce6/CpG@Lip-TD over 7 days.

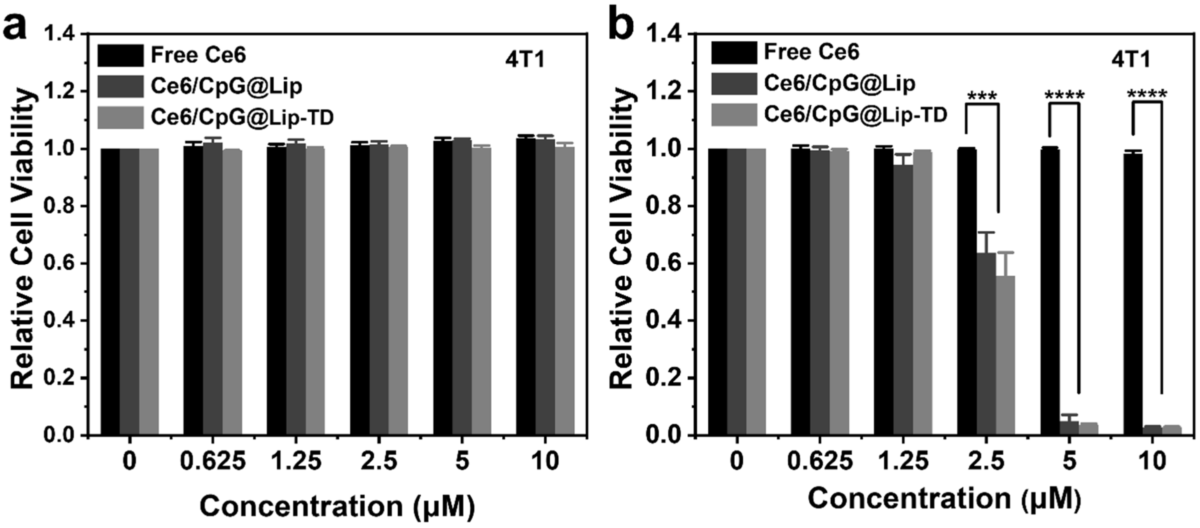

Supplementary Fig.7. Cytotoxicity of Ce6/CpG@Lip-TD. a,b) Relative viabilities of 4T1 cells incubated with free Ce6, Ce6/CpG@Lip and Ce6/CpG@Lip-TD for 4 h, then irradiated **(a)** without or **(b)** with a 660 nm laser for 10 min followed by additional 20 h incubation before the standard MTT assay (mean ± SD, the scale were based on three independent experiments, analyzed by a two-tailed unpaired Student's *t* test, $***P < 0.001$, $****P < 0.0001$)

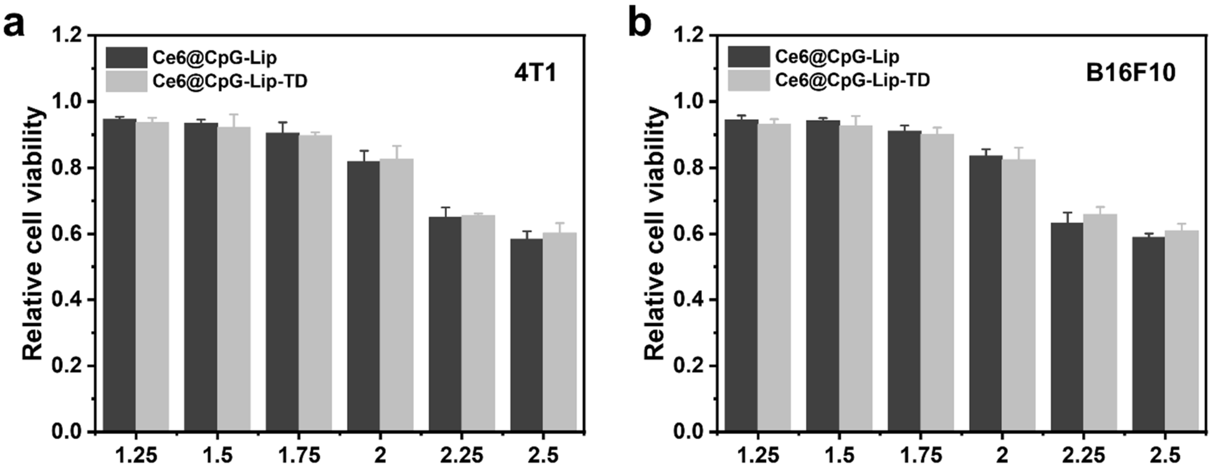

Supplementary Fig.8. Cytotoxicity of Ce6/CpG@Lip-TD for concentrations ranging from 1.25 to 2.5 μM in a)4T1 cell models and b)B16F10 cell models.

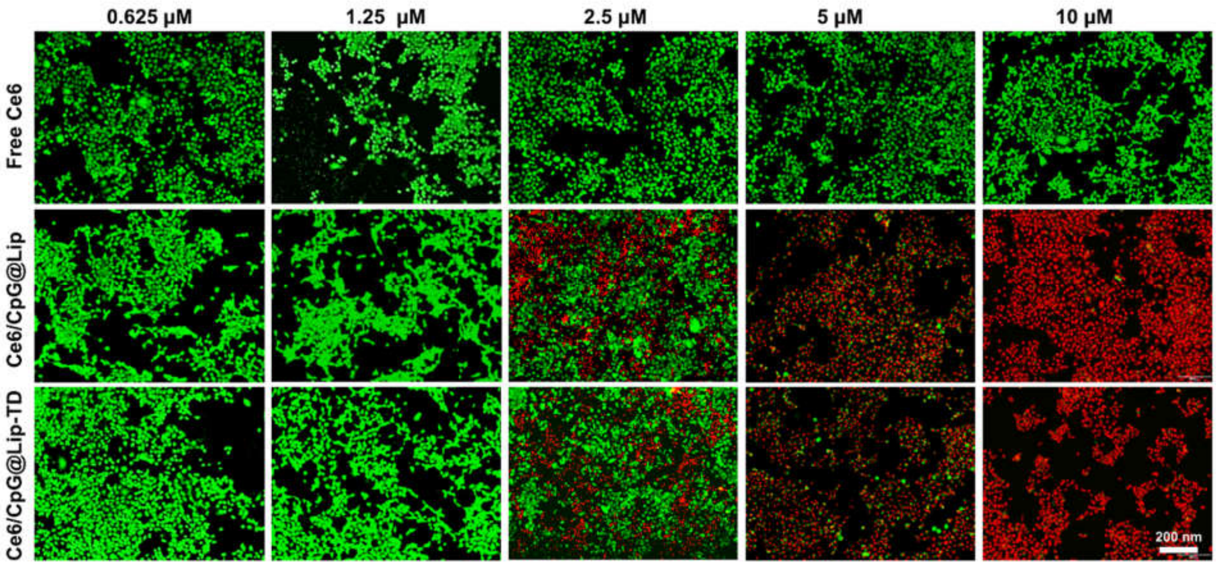

Supplementary Fig.9. Calcein-AM/PI co-staining fluorescence images of B16F10 cells incubated with free Ce6, Ce6/CpG@Lip and Ce6/CpG@Lip-TD under irradiation by 660 nm laser (5 mW cm^{-2}) for 10 min at different Ce6 concentrations.

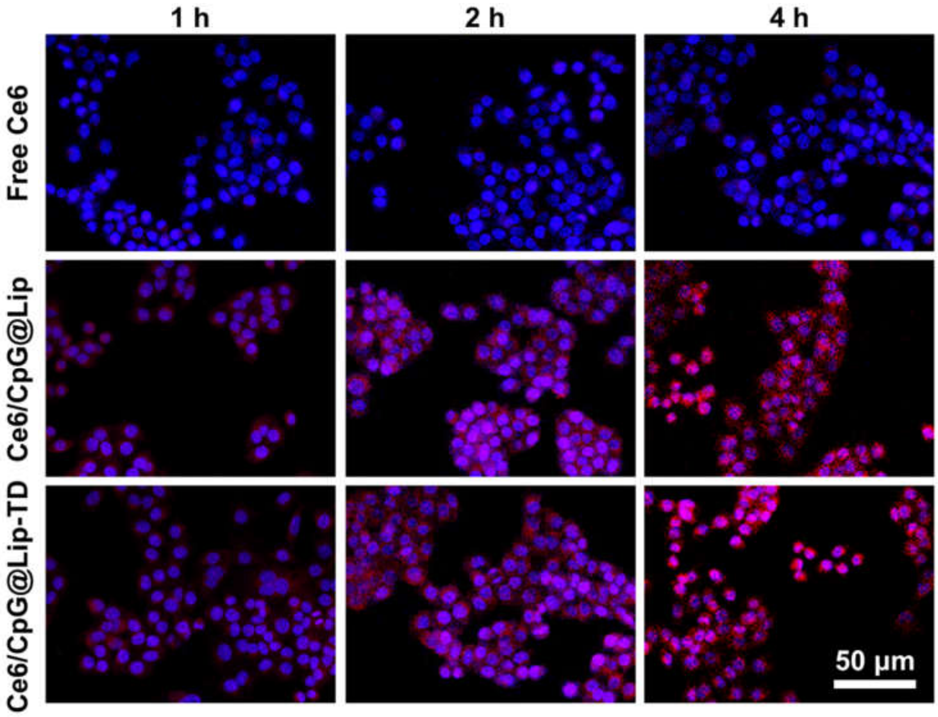

Supplementary Fig.10. CLSM observation of intracellular internalization profiles of Free Ce6, Ce6/CpG@Lip and Ce6/CpG@Lip-TD after incubation with 4T1 cells. DAPI and Ce6 were

excited at 404 nm and 488 nm respectively.

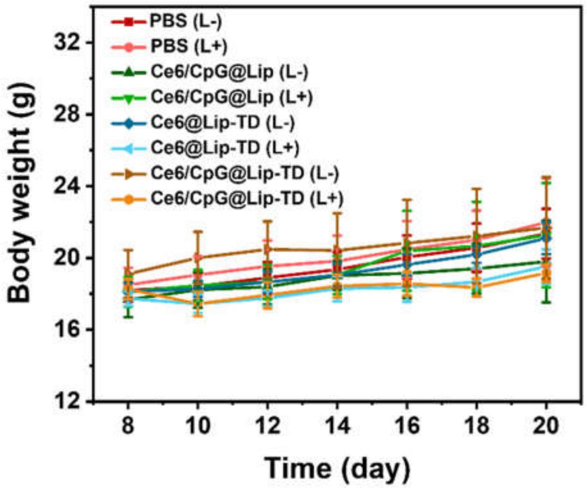

Supplementary Fig.11. The body weight changes of B16F10 tumor mouse models in different treatment groups.

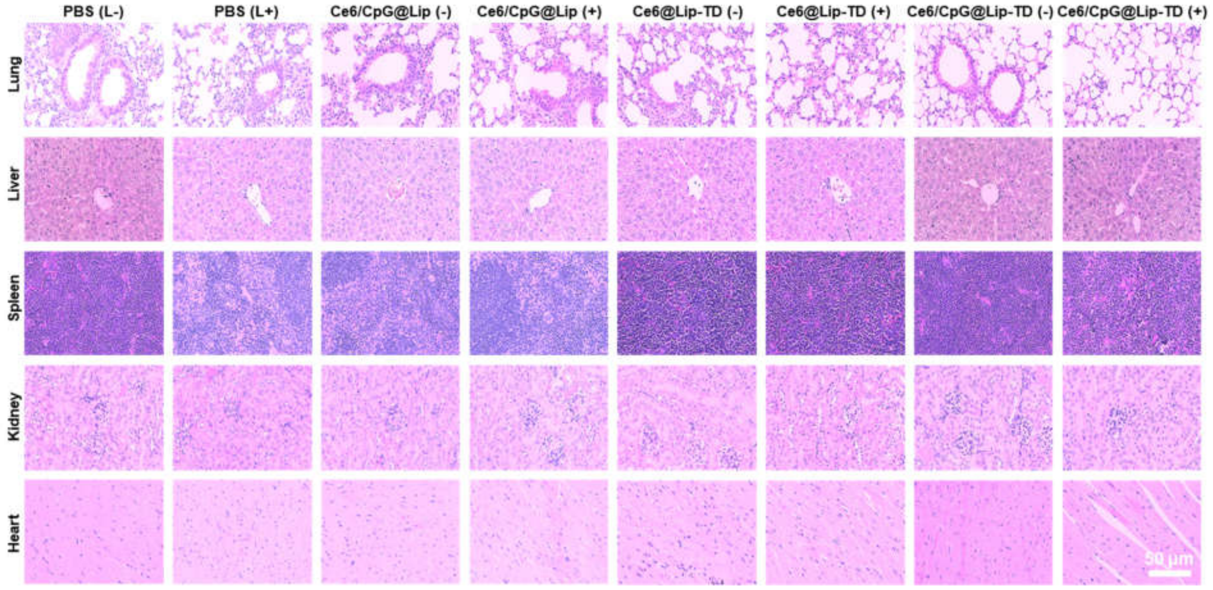

Supplementary Fig.12. Representative H&E-stained histological sections of organs from tumor-bearing mice.

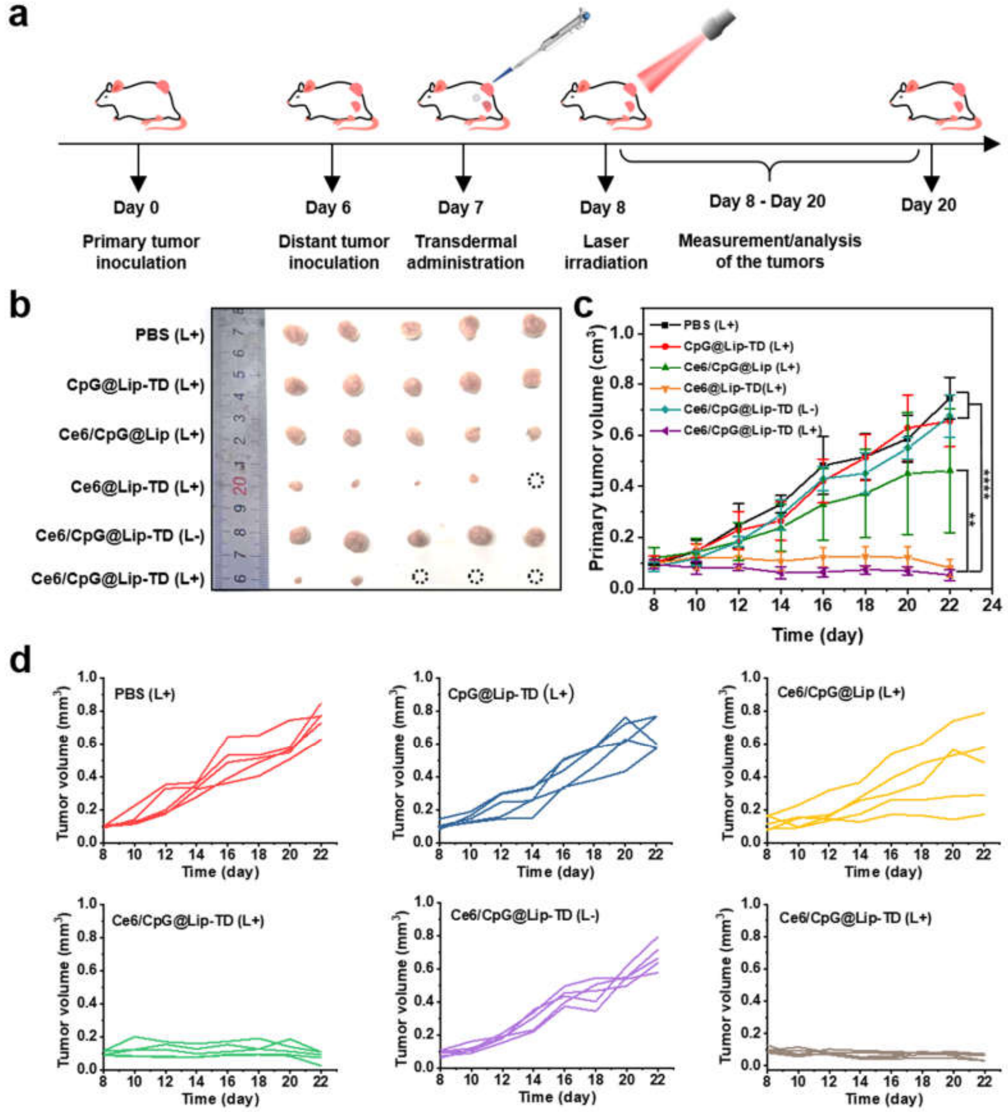

Supplementary Fig. 13. a) The schematic outline shows the in vivo experimental design using 4T1 tumor-bearing mice. **b)** Time-dependent growth curves of primary 4T1 tumors. **c)** Photographs of primary 4T1 tumors isolated from different groups. **d)** Individual 4T1 tumor growth curves of the primary tumors receiving various treatments. (n = 5 biologically independent animals, mean ± SD, analyzed by a two-tailed unpaired Student's t test, $^{**}P < 0.01$; $^{***}P < 0.001$).

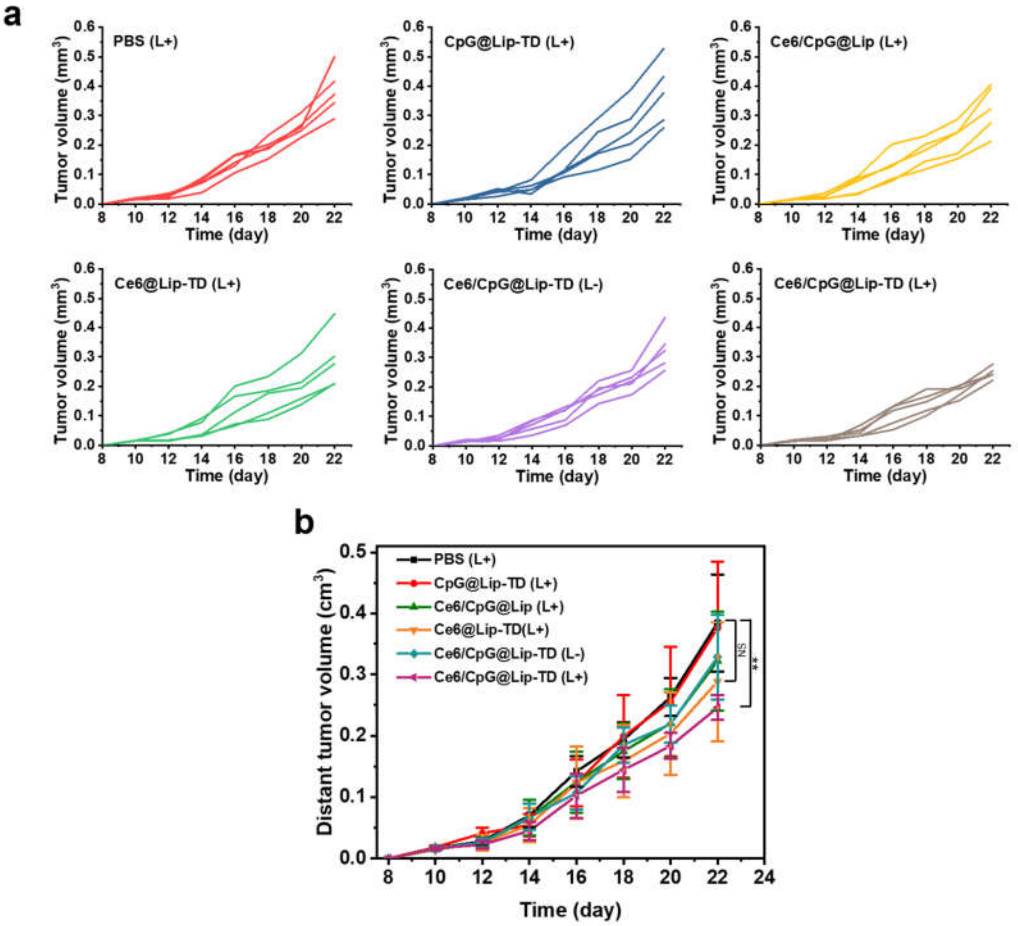

Supplementary Fig.14. a) Individual 4T1 tumor growth curves of the distant tumors receiving various treatments. **b)** Time-dependent growth curves of distant 4T1 tumors. (n = 5 biologically independent animals, mean ± SD, analyzed by a two-tailed unpaired Student's t test, NS (not significant) $^{**}P < 0.01$).

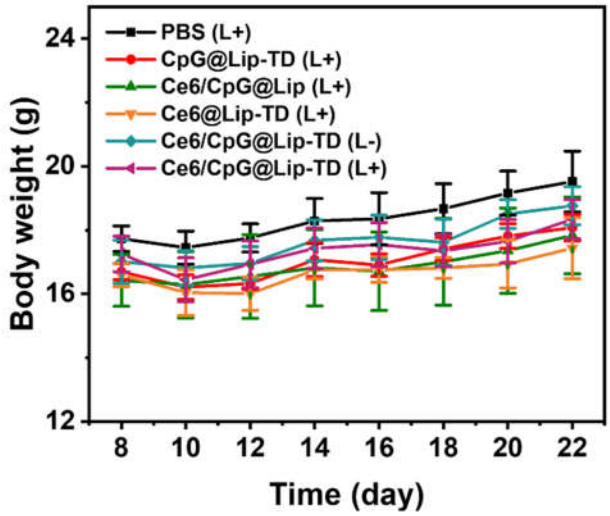

Supplementary Fig.15. The body weight changes of 4T1 tumor mouse models in different treatment groups.

2. Supplementary Tables (S1-S2)

Supplementary Table 1. Characteristics of liposomes.

Classification	Average size [nm]	Zeta-p [mV]	Encapsulation effiency of Ce6 [%]
Free CpG	/	-6.96±0.54	/
Ce6@Lip-TD	113.8±46.23	34.7±2.72	/
Ce6/CpG@Lip	121.6±50.49	28.3±2.22	93.7±2.16%
Ce6/CpG@Lip-TD.	125.0±43.66	26.1±2.04	94.3±2.87%

Supplementary Table 2. Reported irradiation intensity of photosensitizers in nanomaterials.

Photosensitizer	Excitation [nm]	Tumor model	Irradiation intensity [mW cm^{-2}]	Reference
Ce6	660	Hela	650	ACS Appl. Mater. Interfaces 2018, 10, 12431−12440
Ce6	671	4T1	500	ACS Appl. Mater. Interfaces 2019, 11, 5791−5803
Ce6	650	U14	259	Biomaterials 252 (2020) 120093
Ce6	660	4T1	150	Adv. Funct. Mater. 2018, 28, 1706310
Ce6	660	4T1	1000	Biomaterials 122 (2017) 188-200
Ce6	660	HN6-LUV	500	Materials & Design 224 (2022) 111403
Ce6	660	HepG2	500	Chemical Engineering Journal 424 (2021) 130536
Ppa	660	KB	200	Acta Biomaterialia 92 (2019) 219–228
PpIX	635	SCC25	1000	British Journal of Cancer (2016) 115, 805–813
